# Effect of Tillage Practices on Soil Properties and Crop Productivity in Wheat-Mungbean-Rice Cropping System under Subtropical Climatic Conditions

**DOI:** 10.1155/2014/437283

**Published:** 2014-08-14

**Authors:** Md. Khairul Alam, Md. Monirul Islam, Nazmus Salahin, Mirza Hasanuzzaman

**Affiliations:** ^1^Soil Science Division, Bangladesh Agricultural Research Institute, Gazipur 1701, Bangladesh; ^2^Tuber Crops Research Centre, Bangladesh Agricultural Research Institute, Gazipur 1701, Bangladesh; ^3^Department of Agronomy, Faculty of Agriculture, Sher-e-Bangla Agricultural University, Dhaka 1207, Bangladesh

## Abstract

This study was conducted to know cropping cycles required to improve OM status in soil and to investigate the effects of medium-term tillage practices on soil properties and crop yields in Grey Terrace soil of Bangladesh under wheat-mungbean-T. *aman* cropping system. Four different tillage practices, namely, zero tillage (ZT), minimum tillage (MT), conventional tillage (CT), and deep tillage (DT), were studied in a randomized complete block (RCB) design with four replications. Tillage practices showed positive effects on soil properties and crop yields. After four cropping cycles, the highest OM accumulation, the maximum root mass density (0–15 cm soil depth), and the improved physical and chemical properties were recorded in the conservational tillage practices. Bulk and particle densities were decreased due to tillage practices, having the highest reduction of these properties and the highest increase of porosity and field capacity in zero tillage. The highest total N, P, K, and S in their available forms were recorded in zero tillage. All tillage practices showed similar yield after four years of cropping cycles. Therefore, we conclude that zero tillage with 20% residue retention was found to be suitable for soil health and achieving optimum yield under the cropping system in Grey Terrace soil (Aeric Albaquept).

## 1. Introduction

Holistic management of arable soil is the key to dealing with the most complex, dynamic, and interrelated soil properties, thereby maintaining sustainable agricultural production systems, the lone foundation of human civilization. Any management practice imposed on soil for altering the heterogenous body may result in generous or harmful outcomes [1, 2]. Unsuitable management practices cause degradation in soil health (depletion of organic matter and other nutrients) as well as decline in crop productivity [3]. Reducing disturbance of soil by reduced tillage influences several physically [4], chemically [5], and biologically [6, 7] interconnected properties of the natural body.

Soil tillage is among the important factors affecting soil properties and crop yield. Among the crop production factors, tillage contributes up to 20% [8] and affects the sustainable use of soil resources through its influence on soil properties [9]. The judicious use of tillage practices overcomes edaphic constraints, whereas inopportune tillage may cause a variety of undesirable outcomes, for example, soil structure destruction, accelerated erosion, loss of organic matter and fertility, and disruption in cycles of water, organic carbon, and plant nutrient [10]. Reducing tillage positively influences several aspects of the soil whereas excessive and unnecessary tillage operations give rise to opposite phenomena that are harmful to soil. Therefore, currently there is a significant interest and emphasis on the shift from extreme tillage to conservation and no-tillage methods for the purpose of controlling erosion process [11]. Conventional tillage practices cause change in soil structure by modifying soil bulk density and soil moisture content. In addition, repeated disturbance by conventional tillage gives birth to a finer and loose-setting soil structure while conservation and no-tillage methods leave the soil intact [12]. This difference results in a change of characteristics of the pores network. The number, size, and distribution of pores again control the ability of soil to store and diffuse air, water, and agricultural chemicals and, thus, in turn, regulate erosion, runoff, and crop performance [13]. Losses of soil organic C (SOC) and deterioration in other properties exaggerated where conventional tillage was employed [14]. With time, conservation tillage, on the other hand, improves soil quality indicators [15] including SOC storage [16].

During the first 4 years of tillage, Rhoton [17] determined a 10% loss of initial soil organic matter content with plough tillage. Mann [18] also estimated the soil organic matter depletion between 16 and 77% caused by the tillage. In most instances, increased levels of tillage or increased tillage periods resulted in reductions of soil carbon. When conventional tillage is converted to conservation tillage, both CO_2_ emissions from soil and N uptake by the crop are reduced. Al-Kaisi [19] reported that reducing tillage significantly decreases SOC loss from soils with high organic matter content. Continuous cultivation for cereal cropping in the major cereal growing areas of Bangladesh leads to lowering the nutritional status of soil in most of the areas. Hence, the depletion of SOC and N concerned has taken place, a problem which needs to be managed through N fixation by the plant. In this situation, leguminous crop such as mungbean can fix N in the range of 30–40 kg N ha^−1^ [20].

Cropping system has an immense effect on physical and chemical soil properties and thereby on crop productivity [21]. Soil fertility often changes in response to land use and cropping systems and land management practices [22]. Intensive cropping promotes high levels of nutrient extraction from soils without natural replenishment. Limited practices of legume, green manure, and jute based cropping patterns have led to depletion of soil organic matter content in soils of Bangladesh [23]. Use of green manure especially legumes in a cropping pattern could help restore crop productivity. The major cereal cropping system of South Asia is rice and wheat grown on the same field but in different seasons during one year. Currently, about 12 million hectares of land in Pakistan, Nepal, India, and Bangladesh use this cropping pattern, accounting for nearly one-fourth of the region's cereal production. After rice, wheat has become an important component of cropping pattern in Bangladesh which is cultivated mostly after* aman* rice (lowland rice grown in the wet season from June to November in Bangladesh and east India). Crop production could be increased by adopting appropriate tillage operation and selecting suitable crops in the cropping pattern including leguminous crops, which demands intensive field research [23, 24]. Whether conservation tillage practice performs better than the long-practiced traditional tillage practices in terms of improvement of edaphic and yield influencing characters of the specific and unearth soil-water-plant ecosystem of the region is still unknown. As the conservation tillage practices have been reported to manipulate soil positively, they could also be a solution of poorly managed soil condition in the region of rice-wheat cropping system. Effect of medium-term tillage practices on soil properties in Grey Terrace soil under wheat-mungbean-T.* aman* (the tall traditional rice, some of which is deep water rice) cropping system has not been reported. The present study, therefore, has been initiated with the following objectives. The specific objective of the study was to observe how many cropping cycles would be required to build up organic matter (OM) in soil and the general objectives were (1) to evaluate the effects of tillage practices on soil hydrophysical properties, (2) to study the effect of tillage practices on the yield performances of wheat-mungbean-T.* aman* cropping system, and (3) to study the medium-term effect of tillage practices on organic matter status of soil.

## 2. Materials and Methods

### 2.1. Study Area

**Figure 1:**
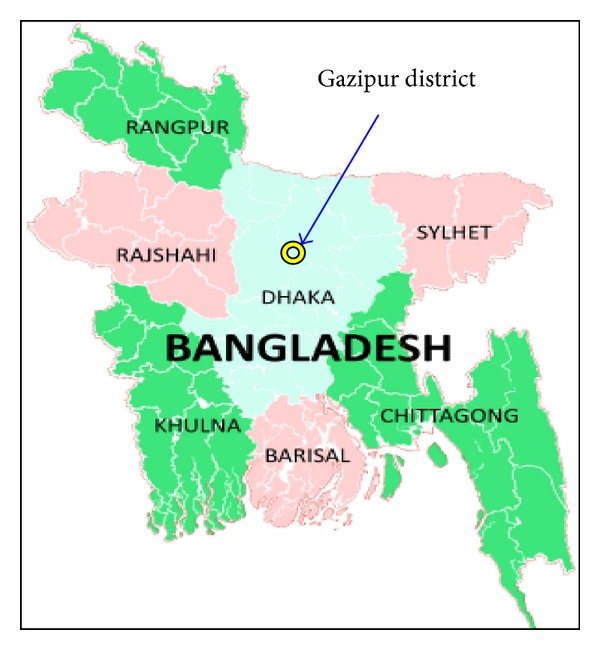
Geographical position of ⊚ Gazipur district (←).

The field experiment was conducted at the Bangladesh Agricultural Research Institute (BARI), Gazipur, Bangladesh, for the four consecutive years from 2008 to 2012. The physical characteristics and chemical status of the initial soil are shown in Tables 2 and 3, respectively. The experimental site is located at the centre of the agroecological zone of Madhupur tract (AEZ-28) at about 24° 23′ north latitude and 90° 08′ east longitude having a mean elevation of 8.4 m above mean sea level. The soil belongs to the Chhiata series of the Grey Terrace soils (Aeric Albaquept) under the order Inceptisols in the USDA Soil Taxonomy [24, 25]. The morphological and taxonomical characteristics of the experimental site are shown in Table 1. The textural class was clay loam having soil pH 5.7 and the land type is medium high. Geographical position of Gazipur district is presented in Figure 1.

**Figure 2:**
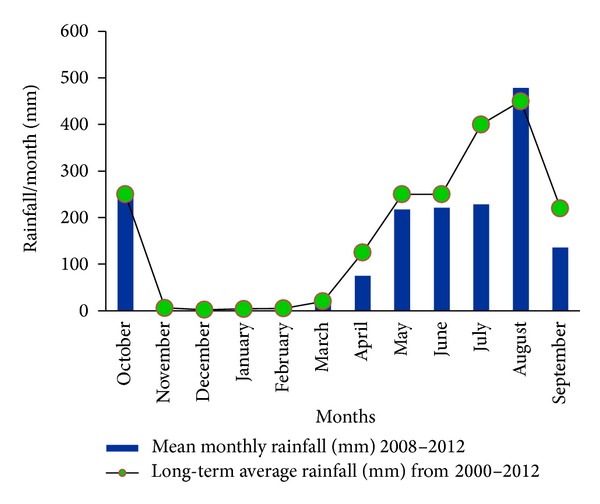
Rainfall (mm) distribution of the experimental site.

**Figure 3:**
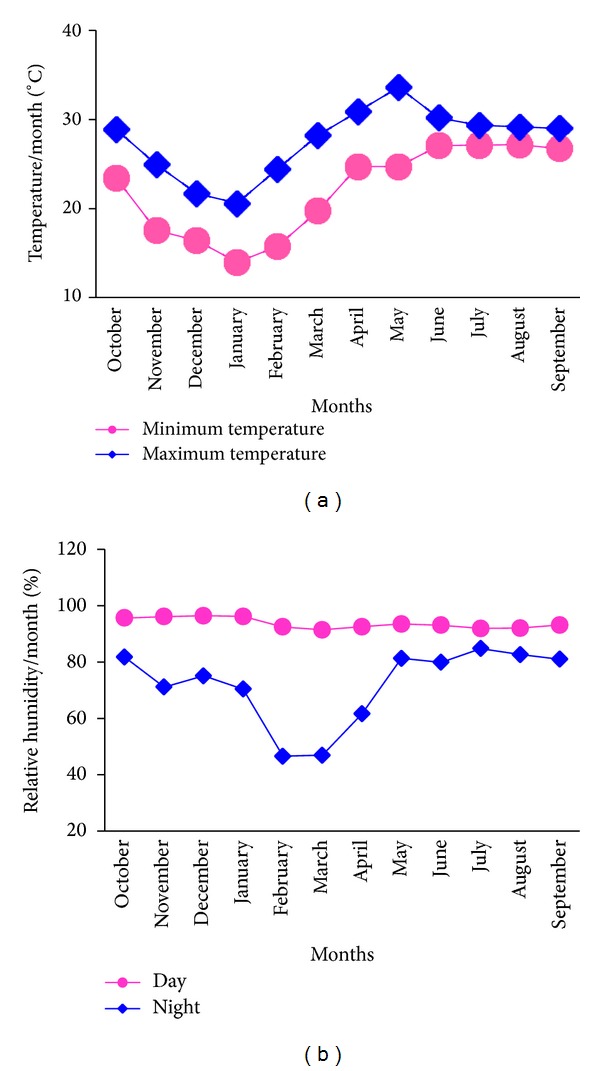
Temperature (a) and relative humidity (b) of the experimental site.

The climate of the experimental area was subtropical, wet, and humid. Heavy rainfall occurs in the monsoon and is scarce in other times. The climatic data of the study area for the period from 2008 to 2012 indicates that the mean annual rainfall is above 1600 mm of which 72.2% is received during the main growing season (Kharif: one of the three seasons in Bengali crop calendar starting from mid-March and stretching to mid-October), that is, from the middle of March 2009 to the middle of October 2009. July and August alone contributed more than 50% to the annual rainfall (Figure 2). From late October to mid-March, the minimum and maximum temperatures were in the lowest range whereas from mid-March onward up to mid-October temperature was in the maximum range. However, the highest maximum temperature was recorded in May (Figure 3(a)).

The periods from October to May are virtually dry. The relative humidity (%) varied between day and night of which at day time relative humidity (%) was about 90 (%) and at night it fluctuated to a wide range from 43 to 85% in February and March, respectively (Figure 3(b)).

### 2.2. Cropping Season

There are three major cropping seasons in Bangladesh, namely, Rabi, Kharif-I, and Kharif-II. Rabi season stretches from the middle of October to the middle of March, Kharif-I season stretches from the middle of March to the end of June, and Kharif-II season stretches from early July to the middle of October. In this experiment, wheat was grown in Rabi season, whereas mungbean and T.* aman* were in the Kharif-I and Kharif-II, respectively.

### 2.3. The Test Crop

The first crop of the cropping system was wheat (*Triticum aestivum* L.) cv. Sourav which was collected from the Wheat Research Centre (WRC) of BARI, Gazipur. It is a semidwarf, early maturing variety having large white grain and is suitable for cultivation in both irrigated and rain-fed conditions. The seeds of mungbean (*Vigna radiata* L. Wilczek) cv. BARI Mung 5 were collected from the Pulse Research Centre of BARI, Gazipur, while seeds of T.* aman* rice (*Oryza sativa* L.) cv. BRRI dhan39 were collected from the Bangladesh Rice Research Institute (BRRI), Gazipur, Bangladesh.

### 2.4. Experimental Design

The experiment was laid out in a randomized complete block design with four replications. The experimental design was performed as follows: zero tillage (ZT: a single slot is opened for seed sowing or transplanting), minimum tillage (MT: ploughed by power tiller maintaining depth by depth control lever up to 6–8 cm), conventional tillage (CT: similar to MT up to 14–16 cm depth), and deep tillage (DT: tillage by chisel plough up to 24–26 cm depth). The unit plot size was 5 m × 4 m.

### 2.5. Fertilizer Application and Other Intercultural Operations

The fertilizer doses for wheat (Sourav), mungbean, and T.* aman* rice were N_120_ P_35_ K_75_ S_20_ Zn_2_, N_20_ P_10_  K_13_ S_5_, and N_90_ P_18_ K_48_ S_7.5_ kg ha^−1^ along with cow dung (CD) 5 t ha^−1^, respectively, based on higher yield goal [25]. The fertilizer requirements were calculated on soil test basis. In the case of first crop (wheat) one third urea, whole amount of triple superphosphate (TSP) and cow dung were applied during final land preparation. The rest of the urea, MoP, gypsum, and ZnSO_4_ were applied in two equal splits at 3rd and 5th weeks after seed sowing. For second crop (mungbean), whole amount of fertilizers was applied during final land preparation. For the third crop (T.* aman* rice), one third of urea and whole TSP were applied during final land preparation and the rest of the urea, MoP, gypsum, and ZnSO_4_ were applied in two equal splits at 3rd and 5th weeks after seedling transplantation. Irrigation and other intercultural operations were done as and when necessary. The soil moisture was monitored intensively with tensiometer and sampling of soil with gravimetric method [26].

### 2.6. Seed Sowing/Transplanting

Wheat (cv. shatabdi) seeds were sown on the last week of November for all the years of experimentation while the first subsequent crop, mungbean (cv. BARI mung 5), was broadcasted by hands on the second week of April and the second subsequent crop, T.* aman* (cv. BRRI dhan 39), was transplanted on the first week of July. After picking pods twice, the total biomass of mungbean was incorporated into soil. The spacing maintained for BRRIdhan 39 and wheat was 25 × 15 cm and 15 × 5 cm, respectively. The experimental plots were kept fixed during the entire growth periods.

### 2.7. Sampling Procedures

In all the cropping years, the wheat was harvested in the first week of April whereas the mungbean harvesting was started in the first week of June and continued up to the third week of June. Likewise, T.* aman* rice was harvested in the first week of November at full maturity. Data of wheat, mungbean, and T.* aman* were recorded from one-square-meter area from each plot and then converted into yield per hectare. All the crops were cut at the ground level. Threshing, cleaning, and drying of grain were done separately plotwise. The weights of grain and straw were recorded plotwise. About twenty percent (20%) residue was retained in experimental field in case of wheat and rice crops. Soil samples were collected at 0–25 cm depth from each plot before sowing/planting and at the end of each cropping cycle in every year.

### 2.8. Soil Analyses

Soil samples were then analyzed for pH, OM, N, P, K, and Zn following standard procedures [5]. Soil pH was measured using a glass electrode pH meter (WTW pH 522) at a soil-water ratio of 1 : 2.5 as described by Ghosh [27], soil organic C was measured by Walkley and Black's wet oxidation method as described by Jackson et al. [28], and total N was measured by micro-Kjeldahl method [5]; available P was determined following the Olsen method [28], exchangeable K was determined using NH_4_OAC extraction method [26], S was determined by turbidimetric method with the help of a spectrophotometer using a wave length of 420 nm [5], Ca was determined by complexometric method of titration using Na_2_-TA as a complexing agent [5], Mg was determined by using NH_4_OAC extraction method [26], and available Zn, Cu, Fe, and Mn were determined by using diethylenetriamine pentaacetic acid (DTPA) extraction method [29]. Particle size distribution was done by hydrometer method [26] and the textural class was determined using the USDA textural triangle. Bulk density and particle density of the soil samples were determined by core sampler method and Pycnometer method, respectively [30]. The soil porosity was computed from the relationship between bulk density and particle density using (1). Soil field capacity and permanent wilting point were measured using pressure plate apparatus, while available water content was calculated using (2) [26]. Consider

(1) $$\begin{array}{c} \text{Porosity}\left(\%\right)=\left(1-\frac{\text{BD}}{\text{PD}}\right)\times 100, \end{array}$$

where BD is bulk density (g cm^−3^), PD is particle density (g cm^−3^), and

(2) $$\begin{array}{c} d=\frac{\text{FC}-\text{PWP}}{100}\times \text{BD}\times \text{Soil}\;\text{depth}, \end{array}$$

where *d* is available water content (cm) at 60 cm depth, FC is field capacity (%), and PWP is permanent wilting point (%).

The double ring infiltrometer method was used to determine the water infiltration and was computed as cumulative infiltration and rate of infiltration in mm h^−1^.

### 2.9. Roots Analyses

The root mass density was measured at maximum vegetative stage in three different soil depths (0–15, 15–30, and 30–45 cm) with auger-like root sampler 15 cm (6 inch) in diameter and 22.5 cm (9 inch) in length using (3) [31]. Consider

(3) $$\begin{array}{c} \text{Root}\;\text{mass}\;\text{density}=\frac{\text{Mass}\;\text{of}\;\text{root}}{\text{Total}\;\text{volume}\;\text{of}\;\text{soil}}\;\text{mg}\;{\text{cm}}^{-3}. \end{array}$$

### 2.10. Statistical Analysis

The analysis of variance for various crop yields and soil physical and chemical properties was performed following ANOVA technique and the mean values were adjudged by Duncan's multiple range test (DMRT) method [32]. Computation and preparation of graphs were done using Microsoft Excel 2003 Program.

## 3. Results

### 3.1. Changes of Soil Physical Properties

#### 3.1.1. Bulk Density, Particle Density, Porosity, Field Capacity, and Permanent Wilting Point

**Figure 4:**
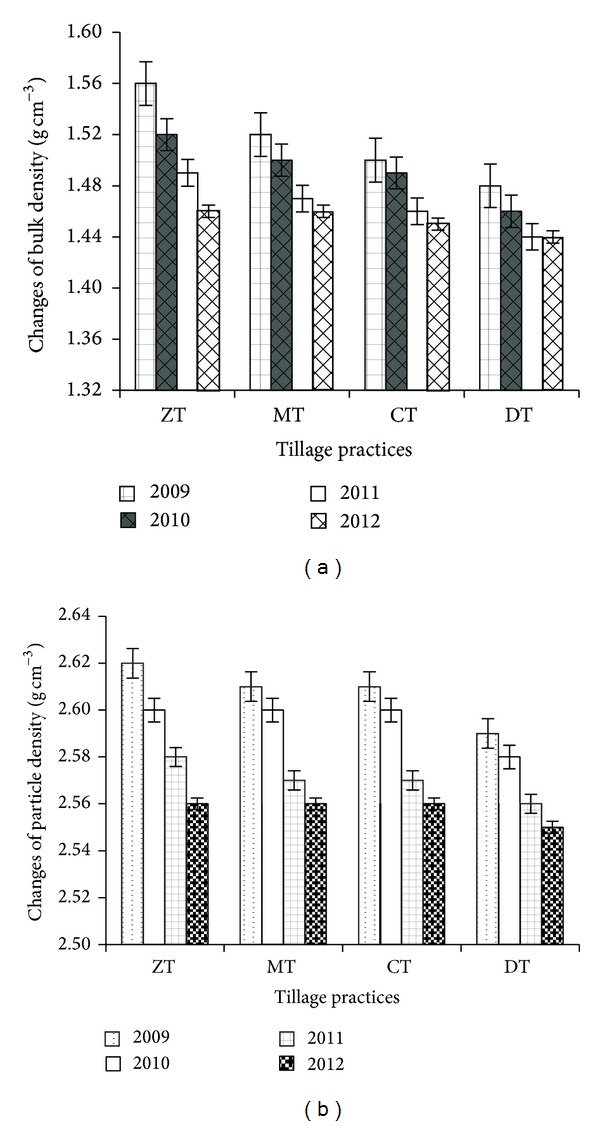
Change in bulk density (a) and particle density (b) as influenced by different tillage practices (most recent year first). Notes: ZT: zero tillage, MT: minimum tillage, CT: conventional tillage, and DT: deep tillage.

**Figure 5:**
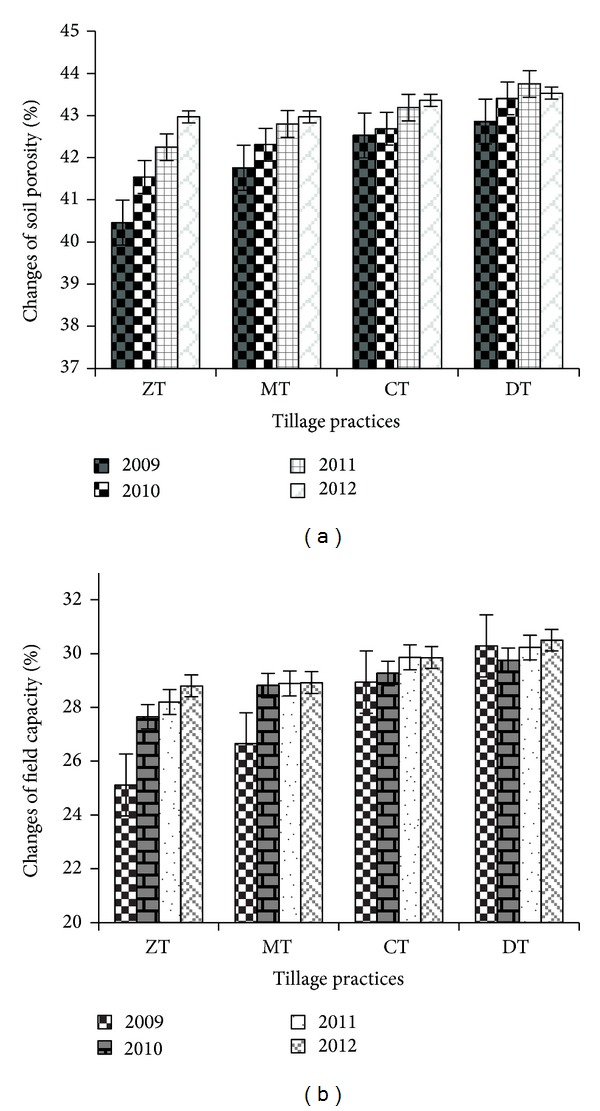
Change in soil porosity (a) and field capacity (b) as influenced by different tillage practices (year most recent first). Notes: ZT: zero tillage, MT: minimum tillage, CT: conventional tillage, and DT: deep tillage. Means ± SE are shown in error bar (*P* ≤ 0.05).

**Figure 6:**
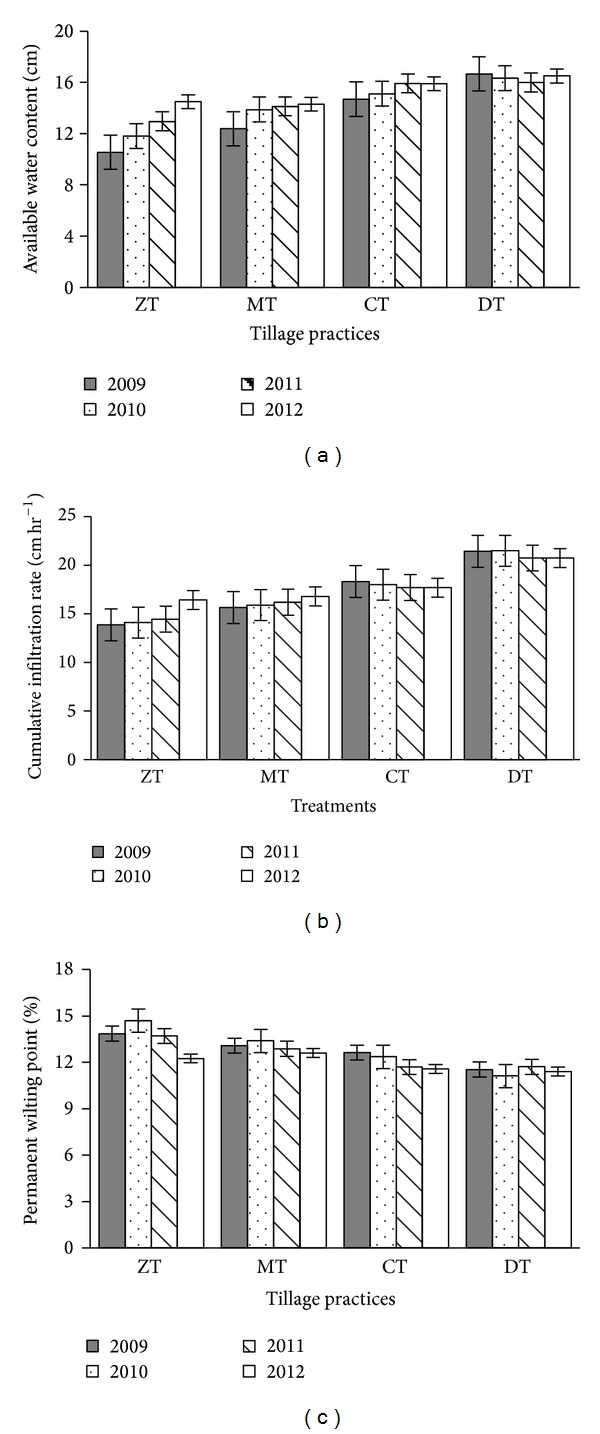
Effect of tillage practice on available water content of soils (a), cumulative infiltration (b), and permanent wilting point (c) (most recent year first). Notes: ZT: zero tillage, MT: minimum tillage, CT: conventional tillage, and DT: deep tillage. Means ± SE are shown in error bar (*P* ≤ 0.05).

Bulk density (Bd), particle density (Pd), porosity, field capacity, and permanent wilting point were influenced by the different tillage practices. Soil bulk density varied considerably (*P* ≤ 0.05) among tillage practices. After four years, bulk density was decreased due to tillage practices. The highest Bd reduction (6.41%) was found in ZT followed by MT (3.95%), while DT showed the lowest reduction (Figure 4(a)). The soil particle density was decreased after four years of study. The highest decrease was noted in ZT and the minimum was in DT (Figure 4(b)). After four years of cropping cycles, porosity was increased from the initial value (6.2, 2.9, and 0.69% increase in ZT, MT, and CT, resp.) (Figure 5(a)). The field capacity (FC) was also increased due to different tillage practices. The highest FC increase (14.65%) was found in ZT followed by MT (8.52%). CT showed the lowest increase of field capacity from the first year value (Figure 5(b)). Permanent wilting point (PWP) was also influenced by the different tillage practices. After four years, the permanent wilting point was decreased due to tillage practices (Figure 6(c)). The highest reduction (11.91%) was found in ZT followed by CT (8.32%) and the lowest reduction (1.13%) in DT.

#### 3.1.2. Soil Water Content

After four years of experimentation, the result showed no significant variation in available water content (AWC) due to different tillage treatments whereas AWCs were significant after completion of the first and second cropping cycles. In the end of the study, maximum available water content (AWC) was found in the deep tillage (16.50 cm) and the minimum AWC (14.30 cm) in ZT (Figure 6(a)).

#### 3.1.3. Infiltration

**Figure 7:**
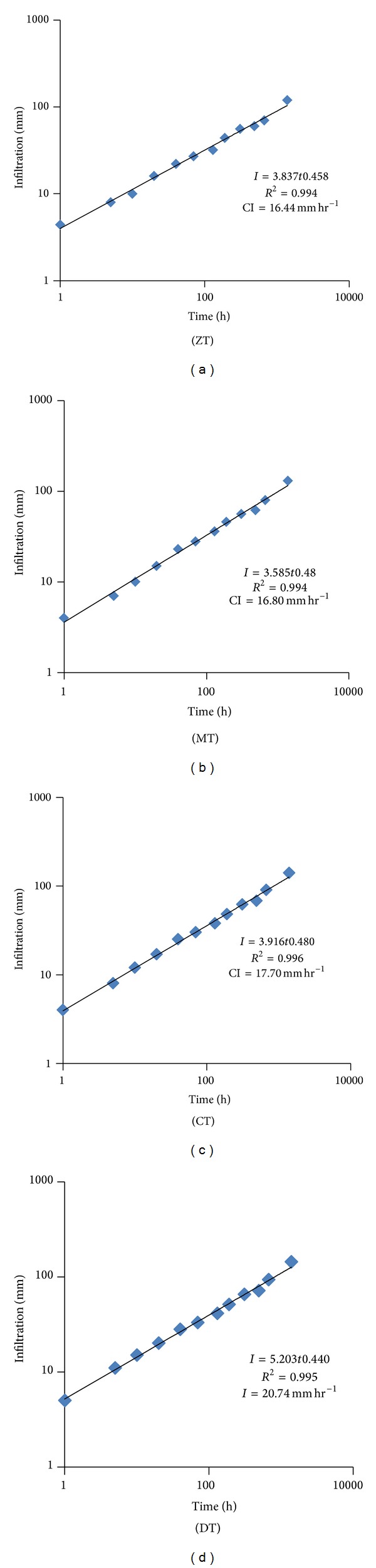
Cumulative infiltration (mm) over cumulative time (hour). Notes: ZT: zero tillage, MT: minimum tillage, CT: conventional tillage, and DT: deep tillage.

Infiltration of water into soil was influenced by different tillage practices. The infiltration rate was found to be increased after every cropping cycle. After four years, the highest increase (18.44%) was found in ZT followed by MT (7.35%) whereas CT and DT showed decreasing trend after two years (Figure 6(b)). The maximum reduction (3.31%) was observed in DT and the minimum was in CT. The highest intercept was found in DT (*K* = 5.203) followed by CT (*K* = 3.92) which explains that deep tillage has higher initial infiltration (Figure 7).

#### 3.1.4. Organic Matter Status of Postharvest Soil

**Figure 8:**
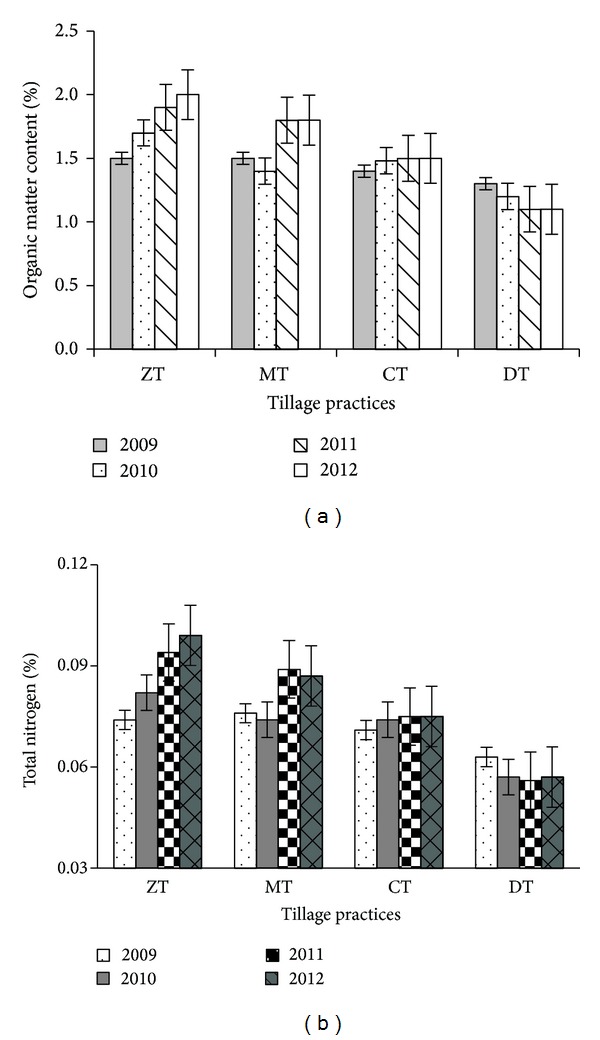
Change in soil organic matter (a) and total nitrogen (%) (b) due to different tillage practices (most recent year first). Notes: ZT: zero tillage, MT: minimum tillage, CT: conventional tillage, and DT: deep tillage. Means ± SE are shown in error bar (*P* ≤ 0.05).

The organic matter content in the initial soil was 1.3% but changed due to different tillage practices after wheat-mungbean-T.* aman* cropping cycles. Organic matter ranged from 1.3 to 1.5% in 2009 and from 1.2 to 1.7% in 2010 (Figure 8(a)) of which the highest OM content of the range (1.7%) was found in ZT and the lowest (1.2%) in DT in both years. In 2011 and 2012, the maximum organic matter content (1.9 and 2.0% in 2011 and 2012, resp.) was recorded in ZT, which was followed by MT (1.8% in 2011 and 2012). DT showed the minimum organic matter (1.1%) (Figure 8(a)). In 2012, the SOM content in ZT was 34.48%, 31.03%, and 25.86% higher than the SOM in 2009, 2010, and 2011, respectively. After four years of experimentation, the SOM content in ZT was 54.76%, 32.00%, and 13.79% greater than the DT, CT, and MT, respectively (Figure 8(a)). It was found that SOM content gradually increased in ZT with increasing time but the reverse is true in the case of DT. After four years, SOM increased by 50% in ZT compared to initial status whereas MT and CT showed comparatively less increment (Figure 8(a)).

### 3.2. The Nutrient Status in Postharvest Soil after Every Cropping Cycle

The nutrient concentrations were significantly variable (*P* ≤ 0.05) among different tillage practices (Table 8 and Figure 8). The total N (%) content ranged from 0.063 to 0.076% in 2009 and from 0.057 to 0.082% in 2010. In 2010, the maximum total N content (0.082%) was found in ZT while MT showed the highest total N (0.076%) in 2009. The minimum total N content (0.063 and 0.057% for 2009 and 2010, resp.) was noted in DT (Figure 8(b)). In 2011 and 2012, ZT showed the highest total N (%) content (0.094 and 0.099% for 2011 and 2012, resp.) followed by MT and the lowest (0.056 and 0.057% for 2011 and 2012, resp.) was in DT. After four years, the total N content was 73.68, 32.0, and 13.79% higher in ZT than the DT, CT, and MT, respectively (Figure 8(b)). It was observed that the total N (%) content gradually increased in ZT and MT with progressing time (Figure 8(b)).

Phosphorus content was also significantly influenced (*P* ≤ 0.05) by the different tillage practices (Table 8). In 2011 and 2012, the highest phosphorus content (18.54 and 20.32 mg kg^−1^ for 2011 and 2012, resp.) was found in ZT which was significantly higher than the other tillage practices. The lowest phosphorus content (13.76 and 14.32 mg kg^−1^) was recorded in DT. The P content was not significantly varied (*P* ≥ 0.05) among different tillage practices in 2009 and 2010. However, it ranged from 12.65 to 13.99 mg kg^−1^ and from 13.21 to 14.96  mg kg^−1^ in 2009 and 2010, respectively. The maximum P content (13.99 and 14.86 ppm for 2009 and 2010, resp.) was detected in ZT and the minimum (12.05 and 13.21 ppm for 2009 and 2010, resp.) was in DT. After four years, the available P was 41.90, 36.74, and 9.66% higher in ZT than the DT, CT, and MT, respectively (Table 8).

Sulphur content was significantly varied (*P* ≤ 0.05) among different tillage practices in all the years. In 2009 and 2010, the highest sulphur (14.00 and 16.12 for 2009 and 2010, resp.) content was found in ZT followed by MT. The lowest S content (12.52 and 13.52 ppm for 2009 and 2010, resp.) was noted in DT (Table 8). In 2011 and 2012, ZT also showed the maximum S content (17.23 and 18.89 ppm for 2011 and 2012, resp.) which was significantly higher than the other tillage practices followed by MT (15.21 and 15.89 ppm for 2011 and 2012, resp.). The lowest S content (14.08 and 14.05 ppm for 2011 and 2012, resp.) was also in DT (Table 8). After four years of experimentation, available S content was 34.45, 30.73, and 18.88% higher in ZT than the DT, CT, and MT, respectively. Potassium content also followed the same trend as N, P, and S. Potassium content was significantly influenced (*P* ≤ 0.05) due to different tillage practices only in 2012. It ranged from 78.0 to 93.61 ppm in 2011 and from 74.1 to 105.3 ppm in 2012. ZT showed the highest concentration of K in all the years and the minimum was in DT (Table 8). After four years of cropping cycles, available K in ZT was 42.11, 35.0, and 17.39% higher than the DT, CT, and MT, respectively (Table 8).

### 3.3. Effect of Tillage on Root Mass Density of Wheat

The root mass density of wheat was measured at three soil depths and variations among (*P* ≤ 0.05) tillage practices at different depths (Table 4) were found. The highest root mass density was found in 0–15 cm depth followed by 15–30 cm depth. The lowest root mass density was noted in 30–45 cm depth (Table 4). In surface soil, ZT showed the maximum root mass density (9.99 mg cm^−3^) followed by MT (9.92 mg cm^−3^). The minimum density was recorded in DT (Table 4). In 30–45 cm depth, the highest root mass density (1.54 mg cm^−3^) was found in DT and the lowest (0.87 mg cm^−3^) was in ZT. As root mass density was highest in the surface soil, tillage effects on the surface would be more important than the deeper layer.

### 3.4. Effect of Tillage on Root Mass Density of Rice

The root mass density of rice was also significantly (*P* ≤ 0.05) influenced by the tillage practices (Table 4). In the surface soil, the root mass density was significantly varied among tillage treatments. The maximum root mass density of 5.40 mg cm^−3^ was recorded in zero tillage. The deep tillage showed the highest root mass density (0.94 mg cm^−3^) in the deeper layer and the lowest (0.49 mg cm^−3^) was in ZT. Among the depths, surface soil showed the maximum root mass density followed by subsurface and the minimum density was noted in the deeper layer. Though the DT showed the highest root mass density in deeper layer, this layer contains very small amount of roots, whereas the maximum root mass density was found in ZT at surface soil where maximum amount of roots was recorded compared to deep layer (Table 4).

### 3.5. Effect of Tillage on the Yield of Wheat

The wheat yield was significantly influenced (*P* ≤ 0.05) by the different tillage practices from 2009 to 2010. The highest grain yield (4.50 and 4.46 t ha^−1^ for 2009 and 2010, resp.) was found in deep tillage followed by CT (4.22 and 4.00 t ha^−1^ for 2009 and 2010, resp.). The lowest grain yield (2.76 and 3.00 t ha^−1^ for 2009 and 2010, resp.) was obtained in ZT (Table 5). The deep tillage also showed the highest straw yield (6.00 and 5.92 t ha^−1^ for 2009 and 2010, resp.) followed by CT (5.50 and 5.80 t ha^−1^ for 2009 and 2010, resp.) and MT (5.10 and 4.60 t ha^−1^ for 2009 and 2010, resp.). The minimum straw was also obtained in ZT. In 2011 and 2012, the wheat grain yield was not significantly varied (*P* ≥ 0.05) among tillage practices. The wheat grain yield ranged from 3.53 to 4.13 t ha^−1^ in 2011 and from 3.69 to 4.11 t ha^−1^ in 2012. After four years, the yield gap was very minimal (negligible) among different tillage practices, though the deep tillage showed the highest yield. In the case of straw yields, a similar trend was found.

### 3.6. Mungbean Yield

Among the four years, mungbean yield was not significantly influenced (*P* ≥ 0.05) by the different tillage practices except for the yield in 2010 (Table 6). After four years (in 2012), the mungbean grain yield ranged from 792 to 820 kg ha^−1^. The highest yield (820 kg ha^−1^) was found in DT followed by ZT (812 kg ha^−1^). The lowest yield (792 kg ha^−1^) was noted in MT (Table 6). It was found that the yield difference was negligible among the tillage practices after four-year cropping cycles.

### 3.7. Effect of Tillage Practices on the Yield of T.* aman*

In 2009 and 2010, the T.* aman* yields were significantly influenced (*P* ≤ 0.05) by the different tillage practices. The grain yield ranged from 2.87 to 4.56 t ha^−1^ in 2009 and from 3.64 to 4.63 t ha^−1^ in 2010. The highest grain yield (4.50 and 4.63 t ha^−1^ for 2009 and 2010, resp.) was found in DT. The minimum grain yield (2.87 and 3.64 for 2009 and 2010, resp.) was recorded in ZT. After two years, rice yield was not significantly variable (*P* ≥ 0.05) among tillage practices (Table 7).

## 4. Discussion

The experiment was conducted during four years indicating that zero and minimum tillage practices had significant influence on soil physical and chemical properties and yields of crops in wheat-mungbean-T.* aman* cropping system, compared to conventional and deep tillage practices.

The bulk density (Bd) was decreased by 9.59, 9.59, 10.34, and 11.11% in DT, CT, MT, and ZT, respectively, compared to initial value. Different tillage practices showed more or less similar influence on bulk density. After the completion of four years, it was found that there was no significant (*P* ≥ 0.05) difference among the different tillage practices. This might be due to the deposition of OM in ZT practice. Soil bulk density is the significant indicator of change of soil physical health and water retention capacity under different tillage depths [33]. A similar result was reported by Sarwar et al. [34]. In New South Wales (NSW), Australia, the soil Bd was reduced by 6.7% in no tillage (at 50 cm depth) compared to conventional tillage after 14 years [35]. He et al. [35] reported that the mean bulk density (in 0–30 cm soil layer depth) under NT and CT treatments was 1.40 and 1.41 Mg m^−3^, respectively, and the difference was negligible in the long terms which is in agreement with the findings of our study. In Chinese Loess Plateau, crop stubble retention under no tillage and controlled traffic has been reported to increase soil organic matter and biotic activity, thereby reducing bulk density in the surface soil layer [35, 36]. Soil organic C has a direct impact on the bulk density or inversely on the porosity of soil, since the particle density of organic matter is considerably lower than that of mineral soil and soil organic matter is often associated with increased aggregation and permanent pore development as a result of soil biological activity [37]. The changes in soil bulk density in 0–0.30 m soil layer are consistent with the porosity results. After 8 years of different management, the mean soil bulk density in 2007 was 0.8–1.5% lower in NT than the CT at Daxing and Changping. The reduced bulk density in NT could be attributed to higher organic matter content [38] and better aggregation [39].

Soil particle density (Pd) was varied insignificantly (*P* ≥ 0.05) among tillage practices after four years of experimentation. For an average between 0 and 25 cm depth, particle density was found to be decreasing in ZT with time (2.56 g cm^−3^) as compared to deep tillage (2.55 g cm^−3^) where particle density was stuck at a fairly constant level. The decrease of Pd in ZT (*P* ≥ 0.05) might be due to accumulation of organic matter (OM) with time. A similar result was also observed by Rühlmann et al. [39] where Pd decreased in top soil and it was related to variation in SOC.

The effects of tillage practices on porosity were smaller but consistently positive over years. After 4 years, porosity was increased in soil from the initial year due to tillage practices. The increase of soil porosity in ZT might be due to the addition of OM and crop residues which was caused by zero and minimum disturbance of soil. Similar results were also reported by He et al. [35]. Many studies have indicated that tillage systems significantly influenced the soil pore size distribution [40]. However, our results were in agreement with the findings of He et al. [35] who reported that total porosity in the 0–15 cm layer was similar under different treatments and also with that of the work of Zhang and Song et al. [40] where no significant difference in porosity was found at the surface layer. Zhu et al. [41] also reported that no tillage with mulch was found to increase 5.5% of total porosity in 0–30 cm soil layer compared to traditional tillage after 4 years.

Soil moisture retentive characteristics (SWR) were varied by tillage practices. Soil water retention (SWR) at field capacity (−33 kPa) was initially higher in the deep tillage (*P* ≥ 0.05) but it was gradually increasing in the soil treated with zero tillage with the advancement of experiment. Plant-available water content gave almost similar result (*P* ≥ 0.05) to soil water retention at FC. Higher difference was also observed in 0–25 cm depth after completion of the first cropping cycle, where AWC was 36.68, 28.18, and 14.78% lower in ZT than the DT, CT, and MT, respectively. After four cropping cycles, the results reflected insignificant AWC among different tillage practices. The increasing trend of water retention in the soil under ZT practices also implied water uptake increase by the crop, resulting in a gradual improvement of crops yield in zero tillage compared to the other tillage practices in the dry season where yields almost remained constant or decreasing in some cases with time. Soils under no-tillage practices have greater water storage capacity than the tilled soils [42]. Fernández-Ugalde [43] conducted an experiment for a medium-term basis and found that the SWR at field capacity was significantly higher in NT than the CT and reported that these differences were particularly noticeable in the soil surface depth where water retention was 23% lower in CT than in NT. In the present study, SWR was found to be increasing in ZT practice with experiment progressing ahead even though the soil water content at field capacity (FC) during the initial year was found to be significantly higher (*P* ≤ 0.05) with DT. In the long run, SWR and AWC would be found to be significantly higher in zero tillage than the other tillage practices as the experiment showed the evidence of OM build-up and other physical characteristics favourable for this. Besides, infiltration is an important soil feature controlling leaching, runoff, and crop water availability [44].

After the first cropping cycle, the variation in soil water infiltration was higher among different tillage practices than the infiltration variation four years apart which was found to be narrowing down (*P* ≥ 0.05). ZT practices promote infiltration and water retention year after year. Schwen et al. [44] reported that soils under no-tillage treatment have greater infiltration rates than the tilled soils. With management for less than a few years, water infiltration in NT may be similar or lower than the CT due to initial compaction and lack of sufficient biological activity for development of stable soil structure [45]. Conservation tillage practice with judicious crop residue management improves aggregate stability [46] and leads to reduced soil detachment and improved infiltration rates [47]. Surface OM is also essential for water infiltration and conservation of nutrients [48]. Wang et al. [49] also reported that conservation tillage may delay run-off by 12–16 min in heavy rainfall and improve final infiltration rate by 60.9% in comparison with conventional mouldboard ploughing in Shanxi province.

The root mass density (RMD) of wheat and rice varied (*P* ≤ 0.05) among the tillage practices and different soil depths. The total RMD of three sampling depths for both crops was found close in range (13.11–11.71 and 7.54–6.87 mg cm^−3^ for wheat and rice, resp.). In 0–15 cm depth, the roots growth was higher in ZT and MT than the CT and DT but the reverse is true in case of subsurface (15–30 cm) and deep soils (30–45 cm). Therefore, ZT plays an important role in root mass density distribution in the soil. The incorporation of biomass from mungbean favoured maximum roots growth [50, 51]. The root mass density was drastically reduced downward, which was associated with the increased soil bulk density in deeper zone. Root proliferation or extensibility was obstructed by the dense or compact layer of the soil profile [52]. Similar results were found by Parker and Lear [53] and Alam and Matin [54] in different crops.

It was observed that the OM content (%) was found to be decreased in deep tillage after each cropping cycle of wheat-mungbean-T.* aman* whereas organic matter was gradually deposited in the soils where no or minimum disturbance occurred throughout the four cropping cycles. A similar result was also found by Chan and Heenan et al. [55] in different tillage practices. Zero tillage along with addition of organic matter and crop residues in the cropping systems has been reported to increase soil organic matter significantly in the 0–25 cm soil layer compared to DT after 4 years. Zhu et al. [41] also observed a similar result where ZT had 4.3% SOM in the 0–30 cm soil layer compared to traditional tillage after 4 years. In addition, improvements of crop yields have been documented where conservation tillage was practiced [56, 57]. Ma and Tong [57] reported that the winter wheat yield in conservation tillage was 10–20% higher than the conventional tillage in Shandong, northern China. Mean wheat yield improvement in no tillage was estimated to be 4.3% between 2003 and 2004 in the more arid Hexi Corridor area of northwest China [58]. In central Texas, United States, after twenty years in wheat cropping system, soil organic matter and total N were increased by 28 and 33% in no tillage at 0–15 cm soil depth [59]. Conservation tillage was also showed to improve soil water content and crop yields in many environments [7, 60], whereas Hammel et al. [60] reported negative effects of no tillage on crop yields in arid areas of the United States. However, frequent and excessive tillage and residue removal in CT and deep tillage by chiseling resulted in significant loss of SOM [61]. Tillage-induced changes in soil organic N are often directly related to changes in SOC. ZT and MT showed significantly (*P* ≤ 0.05) higher concentrations of available N in the surface soil. Soil available P was also significantly (*P* ≤ 0.05) improved by the MT and ZT, particularly in 0–25 cm soil depth. The accumulation of P at the topsoil in ZT and MT can be explained by the limited downward movement of particle-bound P in no-till and minimum-till soils and the upward movement of nutrients from deeper layers through uptake by roots [62]. Roldan et al. [62] observed that SOM increased by up to 15% through no tillage and minimum tillage at 0–50 mm soil depth in Mexico. The significant increases of available N and P in conservation tillage practices were also consistent with the findings of other researchers [63, 64]. In a study, Reyes et al. [64] reported that soil organic carbon (SOC) was higher in NT (2.77% in 0–15 cm depth) compared to CT (2.22% in 0–15 cm depth). Reicosky et al. [65] also reported that SOM content was increased under conservation tillage practices following the accumulation rate from 0 to 1.15 t C ha^−1^ yr^−1^ with the highest values in temperate climatic condition. Similar data were also observed by Lal et al. [66] where organic carbon accumulation rate ranged from 0.1 to 0.5 t ha^−1^ yr^−1^. This aspect is very important due to the multiple roles played by the organic matter in the soil. It regulates biological, physical, and chemical processes that collectively determine soil health.

After four years of experimentation, it was found that there was no difference in grain yield of rice as influenced by DT and ZT. This might be due to the build-up of organic matter in the zero tillage practice which occurred with the progress of cropping cycles. In the present study, the improved soil chemical and physical properties were probably responsible for the increased crop yields in conservation tillage practices (ZT and MT) in Grey Terrace soil under wheat-mungbean-T.* aman* cropping system. As reported by Liao et al. [67] and Xue et al. [68], conservation tillage practices have been shown to increase crop yield considerably.

## 5. Conclusions

After four years, different tillage practices showed that they influenced soil physical and chemical properties along with the improvement of SOM status under wheat-mungbean-T.* aman* cropping systems. ZT with mungbean biomass and residue incorporation conserved moisture in the soil profile and improved other soil properties, reduced the bulk density, and increased OM, porosity, AWC, and RMD. After four years, the chemical properties were also improved due to ZT and MT practices. The highest total N (%), P, K, and S in their available forms were found in zero tillage. All tillage practices showed statistically similar yield after four years of cropping cycles. Therefore, zero tillage (minimum soil disturbance) with 20% residue retention was found to be suitable to improve soil conditions and to achieve optimum yield under wheat-mungbean-T.* aman* cropping system in the Grey Terrace soil (Aeric Albaquept).

## Figures and Tables

**Table 1:** Morphological and taxonomical characteristics of the experimental site.

Morphological characteristics	
Locality	BARI, Gazipur, Bangladesh
Geographic position	24°-0′N latitude, 90°-25′E longitude, 8.40 m height above the sea level
AEZ	Madhupur tract (AEZ 28)
General soil type	Near neutral soil pH, Grey Terrace soils (Aeric Albaquept)

Taxonomic soil classification	
Order	Inceptisol
Suborder	Aquept
Subgroup	Aeric Albaquept
Soil series	Chhiata
Physiographic unit	Madhupur tract
Drainage	Moderate
Flood level	Above flood level
Vegetation	Clean cultivation and maintaining cropping pattern
Topography	Medium high land, 8.40 m height above the sea level

**Table 2:** Physical characteristics of the initial soil of the experimental plot.

Particle size distribution	Value
Sand (%)	35.30
Silt (%)	37.29
Clay (%)	27.41
Textural class	Clay loam
Bulk density (g cm^−3^)	1.60
Particle density (g cm^−3^)	2.58
Total porosity (%)	37.98
Moisture content at field capacity (%)	24.00

**Table 3:** Chemical status of the initial soil of the experimental plot.

Depth	pH	OM	Total *N*	P	S	B	Cu	Fe	Mn	Zn	K	Ca	Mg
(cm)	—	(%)	(%)	(mg kg^−1^)									
0–25	5.7	1.30	0.085	13	12	0.15	7.34	590	17.63	2.12	70	1202	240
Critical level			—	14	14	0.20	1.0	10.0	5.00	2.00	78	400	96

**Table 4:** Effect of tillage practice on root mass density of wheat and rice.

Treatments	Root mass density (mg cm^−3^)							
	Wheat				Rice			
	0–15 cm	15–30 cm	30–45 cm	Total	0–15 cm	15–30 cm	30–45 cm	Total
ZT	9.99	1.98^c^	0.87^b^	12.84	5.40^a^	0.98^b^	0.49^b^	6.87
MT	9.92	2.26^b^	0.93^b^	13.11	4.90^b^	1.26^b^	0.63^b^	6.79
CT	8.72	2.87^ab^	1.21^b^	12.80	4.75^b^	1.87^a^	0.81^a^	7.43
DT	7.21	2.96^a^	1.54^a^	11.71	4.64^b^	1.96^a^	0.94^a^	7.54
SE (±)	0.89	0.19	0.07	—	0.09	0.18	0.06	—

Figures in a column having common letter(s) do not differ significantly at 5% level of DMRT.

ZT: zero tillage, MT: minimum tillage, CT: conventional tillage, and DT: deep tillage.

**Table 5:** The yield of wheat as influenced by different tillage practices.

Treatment	Grain yield (t ha^−1^)				Straw yield (t ha^−1^)			
	2009	2010	2011	2012	2009	2010	2011	2012
ZT	2.76^b^	3.00^b^	3.53	3.69	4.45^c^	3.99	4.22	4.38
MT	3.89^a^	3.88^a^	3.71	3.78	5.10^bc^	4.60	4.8	4.60
CT	4.22^a^	4.00^a^	3.88	3.95	5.50^ab^	5.80	4.91	5.00
DT	4.50^a^	4.46^a^	4.13	4.11	6.00^a^	5.92	5.31	5.34
SE (±)	0.19	0.20	0.39	0.25	0.25	0.60	0.41	0.51

Figures in a column having common letter(s) do not differ significantly at 5% level of DMRT.

ZT: zero tillage, MT: minimum tillage, CT: conventional tillage, and DT: deep tillage.

**Table 6:** Effect of tillage practice on the yield of mungbean.

Treatment	Grain yield (kg ha^−1^) 2009	Biomass yield (t ha^−1^) 2009	Grain yield (kg ha^−1^) 2010	Biomass yield (t ha^−1^) 2010	Grain yield (kg ha^−1^) 2011	Biomass yield (t ha^−1^) 2011	Grain yield (kg ha^−1^) 2012	Biomass yield (t ha^−1^) 2012
ZT	632	6.26	644^c^	6.39^b^	780	7.26	812	7.56
MT	784	7.12	841^b^	6.83^b^	785	7.28	792	7.60
CT	837	7.82	1000^a^	7.68^a^	800	7.62	800	7.87
DT	882	8.15	1100^a^	8.29^a^	820	8.00	820	8.10
SE (±)	12.56	0.14	10.11	0.12	12.11	0.13	11.57	0.13

Figures in a column having common letter(s) do not differ significantly at 5% level of DMRT.

ZT: zero tillage, MT: minimum tillage, CT: conventional tillage, and DT: deep tillage.

**Table 7:** The yield of T. *aman * as influenced by different tillage practices.

Treatment	Grain yield (t ha^−1^)				Straw yield (t ha^−1^)			
	2009	2010	2011	2012	2009	2010	2011	2012
ZT	2.87^c^	3.64^b^	4.18	4.49	3.07^b^	3.75	3.95	4.54
MT	3.77^b^	3.84^ab^	4.24	4.30	4.13^a^	3.96	3.96	4.32
CT	4.40^a^	4.29^ab^	4.29	4.37	4.68^a^	4.39	4.40	4.40
DT	4.56^a^	4.63^a^	4.43	4.51	4.84^a^	4.69	4.60	4.60
SE (±)	0.13	0.28	0.17	0.12	0.27	0.31	0.52	0.11

Figures in a column having common letter(s) do not differ significantly at 5% level of DMRT.

ZT: zero tillage, MT: minimum tillage, CT: conventional tillage, and DT: deep tillage.

**Table 8:** Effect of tillage practice on available P, available K, and available S after wheat-mungbean-T. *aman* cropping sequence.

Treatment	P (ppm)				K (ppm)				S (ppm)			
	2009	2010	2011	2012	2009	2010	2011	2012	2009	2010	2011	2012
ZT	13.99^a^	14.96^a^	18.54^a^	20.32^a^	89.7	89.7	93.6	105.3^a^	14.00^a^	16.12^a^	17.23^a^	18.89^a^
MT	13.97^a^	14.13^a^	15.43^b^	18.53^b^	89.7	85.8	81.9	89.7^b^	13.56^ab^	15.27^ab^	15.21^b^	15.89^b^
CT	13.21^a^	13.92^a^	14.90^b^	14.86^c^	85.8	78.0	81.9	78.0^c^	13.32^ab^	14.23^bc^	14.43^b^	14.45^b^
DT	12.65^a^	13.21^a^	13.76^b^	14.32^c^	85.8	78.0	78.0	74.1^c^	12.52^b^	13.52^c^	14.08^b^	14.05^b^
SE (±)	0.95	1.09	0.61	0.27	8.08	5.69	5.39	2.06	0.36	0.43	0.44	0.65

Figures in a column having common letter(s) do not differ significantly at 5% level of DMRT.

ZT: zero tillage, MT; minimum tillage, CT: conventional tillage, and DT: deep tillage.

## Abbreviations

AEZ: Agroecological zone
ANOVA: Analysis of variance
B: Boron
BARC: Bangladesh Agricultural Research Council
BD: Bulk density
BRRI: Bangladesh Rice Research Institute
CD: Cow dung
Cu: Copper
DAS: Days after sowing
DAT: Days after transplanting
DMRT: Duncan's multiple range test
DTPA: Diethylenetriamine pentaacetic acid
E longitude: East longitude
FC: Field capacity
Fe: Iron
FRG: Fertilizer recommendation guide
PD: Particle density
RDF: Recommended dose of fertilizer
SOC: Soil organic carbon
S: Sulphur
T.* aman*: Transplanted* aman*
t: Ton
TSP: Triple superphosphate
RMD: Root mass density
SWR: Soil water retention.
